# Isolation and Genotyping of Adenoviruses from Wastewater and Diarrheal Samples in Egypt from 2016 to 2020

**DOI:** 10.3390/v14102192

**Published:** 2022-10-04

**Authors:** Abdou Kamal Allayeh, Sahar Abd Al-Daim, Nehal Ahmed, Mona El-Gayar, Ahmed Mostafa

**Affiliations:** 1 Virology Lab 176, Water Pollution Research Department, Environment and Climate Change Institute, National Research Centre, Dokki, Giza 12622, Egypt; 2 Microbiology Department, Faculty of Pharmacy, Ain Shams University, El-Qobba Bridge, Cairo 11566, Egypt; 3 Center of Scientific Excellence for Influenza Viruses, National Research Centre, Dokki, Giza 12622, Egypt

**Keywords:** *Adenoviruses*, isolation, genotyping, stool, sewage, Egypt

## Abstract

Human adenoviruses (HAdV) are a prevalent cause of diarrhea in children all over the world. Adenoviral infections are responsible for 2% to 10% of diarrheic cases. A long-term investigation was required to gain better knowledge about the incidence of HAdV in Egypt. Herein, we conducted 5 years of detection, isolation, and genotyping of HAdV in fecal and sewage samples from 2016 to 2020, in Cairo, Egypt using molecular and cell culture assays. Human adenoviruses were identified in 35 of 447 fecal samples (7.8%), but only 53.3% (64/120) of the sewage samples. Children under the age of two had the highest positive rate for HAdV infection (77.1%). Species F of HAdV was the most common prevalent genotype in fecal and sewage samples, at 88.5% and 85.9%, respectively. The most prevalent genotypes detected in fecal samples were HAdV-41 (71.2%), HAdV-40 (17.2%), HAdV-6 (5.7%), and HAdV-1 (5.7%). In contrast, the most common genotypes in sewage samples were HAdV-41 (64%), HAdVs-40 (21.8%), HAdV-6 (7.8%), HAdV-1 (4.7%), and HAdV-2 (1.6%). HAdV was detected in all months of the year, with a peak period for clinical samples from December to February (*p* < 0.001), which matched Egypt’s rainy season, while the monthly distribution of HAdV in sewage samples remained consistent throughout the year, with no statistically significant peak period. Interestingly, the HAdV-type 41 genotype was the most common genotype during all of the years of this study. Throughout a 5-year period, our work revealed the infection rate, seasonal distribution, virus isolates, and genetic diversity of HAdV infections in environmental and clinical samples in Cairo, Egypt. Non-enteric adenovirus types (1, 2 and 6), as well as enteric adenovirus (41 and 40), may play a key role in gastroenteritis in Egypt.

## 1. Introduction

Adenoviruses (HAdV) are a leading pathogen of clinical diseases, such as gastroenteritis, conjunctivitis, respiratory illnesses, hemorrhagic cystitis, and systemic infections [1,2,3]. Mastadenovirus, aviadenovirus, atadenovirus, siadenovirus, ichtadenovirus, and testadenovirus are the six genera of the *Adenoviridae* family that have been classified to date. Within the *Adenoviridae* family, human adenoviruses have been categorized into seven species (A, B, C, D, E, F, and G) and have more than 110 types [4,5,6]. Adenovirus infections cause pathogenesis in a variety of human organs. Adenoviruses B and C are the most common respiratory infections, with Adenoviruses species A impacting the respiratory system in immunocompromised patients, and Adenoviruses F, including types 40 and 41, have been identified as one of the principal viruses causing infantile gastroenteritis [7]. Epidemiological investigations of HAdV undertaken in numerous countries throughout the world, including India, Bangladesh, Brazil, Korea, and China, revealed that the virus infected 2% to 10% of diarrheic patients, with the virus mostly infecting infants under the age of two [8,9,10,11]. In Egypt, just a few studies show the incidence of enteric adenovirus infections in children [12,13,14,15]. 

In both developed and developing nations, norovirus is considered the second main cause of viral acute gastroenteritis after rotavirus [16]. The reported rates of rotavirus, norovirus, and adenovirus infections in Egypt varied from 31% to 91.4%, 13.48% to 26%, and 6.7% to 84%, respectively [12,14,17,18]. The detection range of enteric adenoviruses makes adenovirus the second main cause of viral acute gastroenteritis in Egypt, behind rotavirus.

Primarily, few reports have documented the identification of HAdV in children with diarrhea, and those reports only focused on the detection of enteric adenoviruses types 40 and 41, and they were not consistently conducted. Here, we conducted a 5-year study detecting, isolating, and genotyping HAdV, circulating in environment and clinical samples in Egypt from 2016 to 2020, to gain a better understanding of the incidence of HAdV infection through long-term investigation.

## 2. Materials and Methods

### 2.1. Patients and Study Design

**Figure 1:**
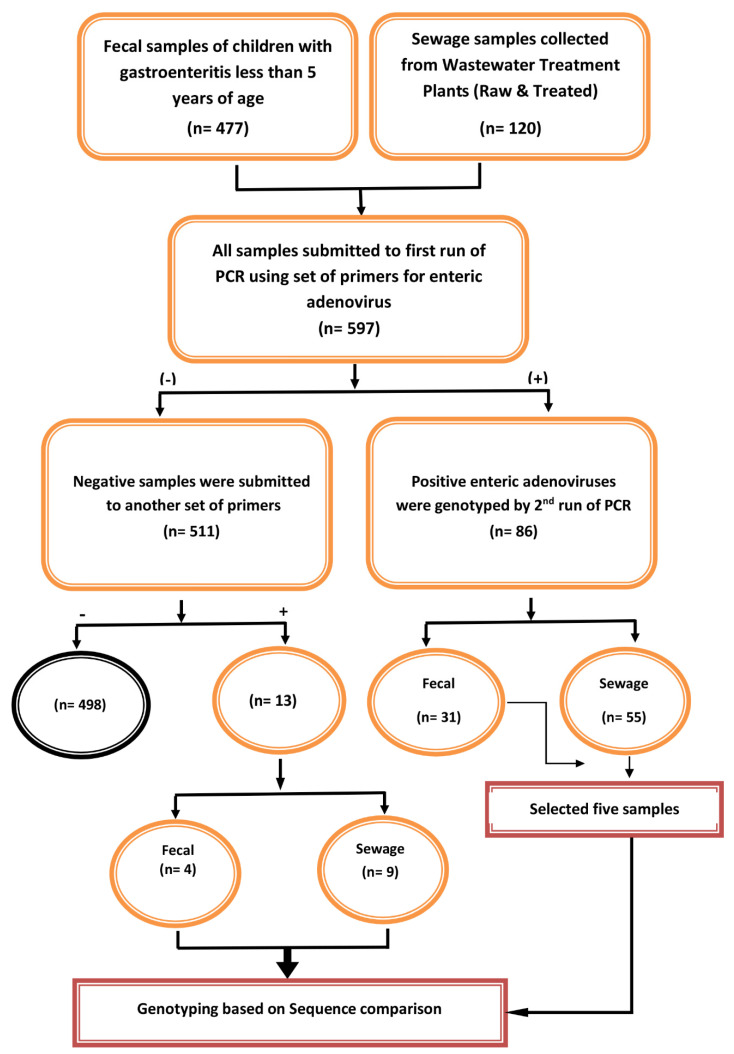
Simplified flow chart describing the number and type of collected samples and their further processing (typing and genotyping) during this study.

The ethical committee of the Faculty of Pharmacy at Ain Shams University in Cairo, Egypt, authorized this study in accordance with the principles of the Helsinki Declaration (ENREC-ASU-2019-83). This study included hospitalized children with severe diarrhea. Acute diarrhea was defined as diarrhea that lasted between 24 h and 14 days. Children who were unable to provide a stool sample on the day of admission were excluded from the study. Children with diarrhea whose parents refused to participate in the research were also excluded. Fecal specimens (*n* = 447) were obtained from children under the age of five who were admitted to Abu El-Reesh Hospital with acute diarrhea, from 2016 to 2020 (Figure 1). The fecal samples were immediately transferred in sterile plastic cups to Egypt’s National Research Centre’s virology lab. Until used, all of the specimens were stored frozen at −80 °C.

### 2.2. Fecal Samples Processing

Fecal specimens were suspended in 10% PBS (pH 7.4). After centrifuging stool suspensions at 8500× *g* for 10 min, DNA was extracted from 140 μL of the supernatant using a Qiagen Viral DNA Extraction Kit (Cat. No., 57704; QIAGEN, Hilden, Germany) according to manufacturer instructions. The DNA was either immediately subjected to a PCR test or kept at −80 °C until needed.

### 2.3. Environmental Samples Collection

Wastewater samples (*n* = 120) were taken from inlet and outlet effluents at the Zinin wastewater treatment facility, Egypt. This wastewater treatment facility had a capacity of 330,000 m^3^, and the final effluent was discharged into the Nile. From 2016 to 2020, samples were collected on a monthly basis. Two liters of each sample were collected in a clean plastic container and delivered to Egypt’s National Research Centre’s virology lab.

### 2.4. Sewage Samples Preparation

According to the USEPA [19], wastewater samples were concentrated. To boost the stability of the viruses in the samples during transit, 2.5 mL of 1 M magnesium chloride per liter of sample was added [20]. By using 1N HCl, the pH of wastewater samples was adjusted to pH 3.5 before filtering using a nitrocellulose membrane filter (0.2 m pore size, 142 mm in diameter) on a filter holder. The adsorbed viruses on the filter were eluted in 100 mL of 3% beef extract—0.05 M glycine solution and re-concentrated as previously described by Katzenelson et al. [21]. The manufacturer’s procedure was followed to extract viral DNA from 140 μL of concentrated samples using a Qiagen Viral DNA Extraction Kit (QIAGEN, Germany). The DNA was either subjected to a PCR test right away or stored at −80 °C for later analysis.

### 2.5. Detection and Genotyping of Adenoviruses by Nested PCR 

For detection of human enteric adenovirus, in accordance with Puig et al. [22], the primers of Hexon gene Forward primer Hex AA1885 (5′- GCCGCAGTGGTCTTACATGCACATC-3′) and Reverse primer Hex1913 (5′- CAGCACGCCGCGGATGTCAAAGT-3′)] were used to amplify 300 bp amplicon. According to Pring-Akerblom and Adrian [23], a second run of amplification was performed for genotyping of enteric viruses as the pervious reaction, except using specific primer sequences as follows: H1 (5′-TTGACATCCGCGGCGTGCTG-3′), Ad40 (5′-TATTCTGAGACCAGTTAGTT-3′), and Ad41 (5′-CTGCAGTCCAGGTTTGGCCA-3′). Another set of primers was used to amplify a conserved 482 bp sequence common to detect all human adenovirus types Ad1 (5′-TTCCCCATGGCTCAYAACAC-3′) and Ad2 (5′-CCCTGGTAKCCRATRTTGTA-3′) [24]. The reaction mixture was heated to 94 °C for 3 min in the Bio-Rad thermal cycler (Model: T100), followed by 35 cycles of 94 °C for 30 s, 55 °C for 1 min, and 72 °C for 1 min. The last had a 5-min extension cycle. A tenth of the PCR master mix (Cat. No. K1071; ThermoFisher Scientific, Waltham, MA, USA) was exposed to a second PCR run under identical circumstances as the first, but with nested primers. After amplification, PCR products (8 μL/sample) were separated by gel electrophoresis in 1.5% agarose. The fragments were seen under UV light after being stained with ethidium bromide.

### 2.6. Isolation of Adenoviruses Using HEp-2 Cell Line

Using 0.01 M PBS, pH 7.0, with 400 U of penicillin per ml and 400 µg of streptomycin per ml, 10% suspensions of all PCR positive adenovirus samples were subjected to isolation and propagation. The suspensions were cleared by centrifugation at 2500× *g* for 10 min and used for the inoculation of cell cultures. The HEp-2 cell line (Catalog no.; CCL23; ATCC) at passage 381 was provided by Holding Company for Biological and Vaccines in Egypt (VACSERA), and used for the isolation of adenoviruses. The HEp-2 was maintained in Dulbecco’s minimal essential medium (DMEM), which included 10% fetal bovine serum, glutamine, 100 units/mL penicillin, and 100 g/mL streptomycin, and pass aged twice a week in a subculture ration of 1:3 at 37 °C in 5% CO_2_ environment. After removing the media from confluent cell monolayers, 100 µL of stool suspension was applied to each well of a 6-well plate with cell density 10^6^/mL. On a rocking platform, the suspension was adsorbed for 1 h at room temperature. The infected cell cultures were then grown in DMEM, which included 5% fetal calf serum, glutamine, 100 units/mL penicillin, and 100 g/mL streptomycin. Each viral isolate was grown in cells at 37 °C in 5% CO_2_ environment. The cells were observed for cytopathic effect (CPE) daily for 6–7 days (Figure S1). Cells were photographed using an inverted microscope (LABOMED, Model: TCM 400) equipped with an industrial digital camera 14 MP. Before being inoculated onto new confluent monolayer cells for onward passage, the infected cells were freeze-thawed three times. Although full CPE is usually seen after the second passage, the absence of CPE after four successive passages was considered negative for Adenovirus isolation. Infected culture with CPE was frozen-thawed three times, centrifuged, and viral supernatants were aliquot and stored at 80 °C [25]. 

### 2.7. Amplification Using qPCR

Using a Maxima SYBR Green Kit (ThermoFisher Scientific, Waltham, MA, USA) with the previously set of primers in PCR [19], the viral titer in samples was quantified according to the manufacturer’s protocol. The real-time reactions were carried out on a QIAGEN Rotor-Gene Q platform for 3 min at 94 °C, followed by 40 cycles of 94 °C for 30 s, 55 °C for 30 s, and 72 °C for 1 min. Amplification data were gathered and examined in real time as an increase in reporter fluorescence. The adenovirus linear standard curve was created by a serial dilution of the HAdV40 hexon gene in a plasmid vector (pCR™4-TOPO™ Vector), using a Topo TA cloning kit (Invitrogen, Carlsbad, CA, USA), as reported by Xagoraraki et al. [26]. Using a one-to-one conversion factor, the amounts of HAdV in environmental and clinical samples were converted from hexon gene copies per liter for environmental samples or gram for clinical samples. 

### 2.8. Sequencing of PCRproducts

An MEGA total fragment DNA purification kit (iNtRON, Hong Kong, China) was used to purify PCR products according to manufacturer instructions. An ABI Prism^®^ 310 Genetic Analyzer was used to retrieve the nucleotide sequences (Colors-Lab, Cairo, Egypt). In summary, 7 µL of fragments were sequenced with the exact primers given in the PCR section using an ABI Prism Big dye termination cycle sequencing kit (Applied Biosystems, Waltham, MA, USA). 

### 2.9. Nucleotide Sequence Accession Numbers 

The GenBank accession numbers for some sequences reported from this study are as follows: ON482338, ON482339, ON482340, ON482341, ON482342, ON482343, ON482344, and ON482345. The acquired hexon gene sequences were aligned and compared to other adenoviruses sequences in the GenBank database (www.ncbi.nlm.nih.gov; accessed on 15 August 2022). 

### 2.10. Statistical Analysis 

For statistical analysis, the GraphPad Prism program v.5.0 was employed. A Chi-square test was used to investigate the relevance of HAdV infection rates across gender and months, as well as to assess differences in viral infection frequencies across various groups. A statistically significant *p*-value of 0.05 was used.

## 3. Results

### 3.1. Incidence of Adenovirus and Viral Load Analysis in Clinical Samples 

HAdV were identified in 35 (7.82%) of 447 hospitalized children’s fecal samples across a 5-year research period. The frequency of HAdV infection was 5.8% in 2016, 7.7% in 2017, 12.8% in 2018, 7.3% in 2019, and 3.9% in 2020 (Table 1). There were 189 girls and 258 boys among the 447 patients, with 5.8% (11/189) of the 35 HAdV-positive samples from females and 9.3% (24/258) from males, with an incidence ratio of (1:2.2), respectively. 

The total detection rates of male and female positive HAdV were comparable and did not significantly differ (*p* = 0.19). Children under the age of two were found to account for 77.1% (27/35) of infected children, a statistically significant difference (*p* < 0.001) (Table 2). HAdV were found in fecal samples in viral titer ranging from 5.5 × 10^5^ to 3.9 × 10^6^ genome copies/gram (mean, 1.7 × 10^6^ genome copies/gram).

### 3.2. Incidence of Adenovirus and Viral Load in Environmental Samples 

Sewage samples were collected on a regular basis from Egypt’s wastewater treatment plant. PCR was used to investigate 120 raw and treated sewage samples. Using specific primers, 64 of 120 samples were found to be positive for adenoviruses (53.3%). Adenoviruses were recovered in larger quantities from inlet effluents than from outflow effluents (*p* < 0.001) (Table 3). HAdV were found in raw sewage in concentrations ranging from 1.8 × 10^3^ to 2.6 × 10^5^ genome copies/liter (mean, 1.2 × 10^4^ genome copies/liter), and in outlet effluent in concentrations ranging from 1.7 × 10^1^ to 3.2 × 10^3^ genome copies/liter (mean, 2.0 × 10^2^ genome copies/liter).

### 3.3. Comprehensive Distribution of Adenoviruses in Clinical and Environmental Samples on a Monthly Basis 

**Figure 2:**
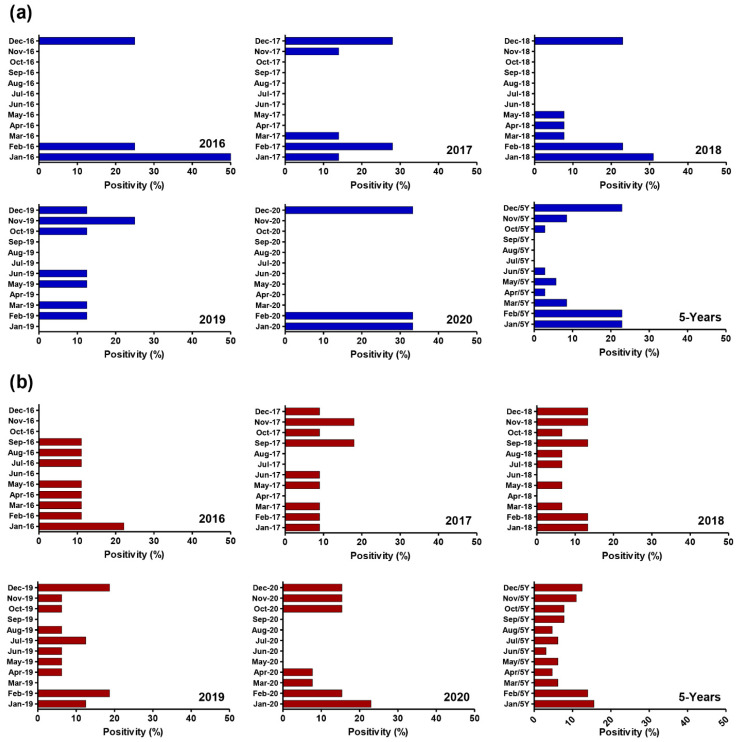
Monthly distribution of human adenovirus infections in both clinical and environmental samples in Egypt from 2016 to 2020. (**a**) Monthly Distribution of HAdV in clinical samples includes collectivity slide for 5-year positivity %; (**b**) Monthly Distribution of HAdV in Environmental samples includes collectivity slide for 5-year positivity %. HAdV levels in clinical samples peak mainly in December, January, and February, although HAds levels in environmental samples are observed throughout the year.

Figure 2 depicts the monthly distribution of HAdV from January 2016 to December 2020. The results indicated HAdV were present all year, with a peak period for clinical samples from December to February (*p* < 0.001), which matched Egypt’s rainy season. The monthly distribution of HAdV in sewage samples remained consistent throughout the year, with no statistically significant peak period. The monthly distribution of HAdV-positive cases, as analyzed over the total 5 years, revealed that the highest rates of HAdV detection were in January [(stool, 8/35, 22.9%) and (sewage, 10/64, 15.6%)], followed by February [(stool, 8/35, 22.9%) and (sewage, 9/64, 14.0%)] and December [(stool, 8/35, 22.9%) and (sewage, 10/64, 12.5%)].

### 3.4. Comprehensive Distribution of Adenovirus Genotypes in Clinical and Environmental Samples

**Figure 3:**
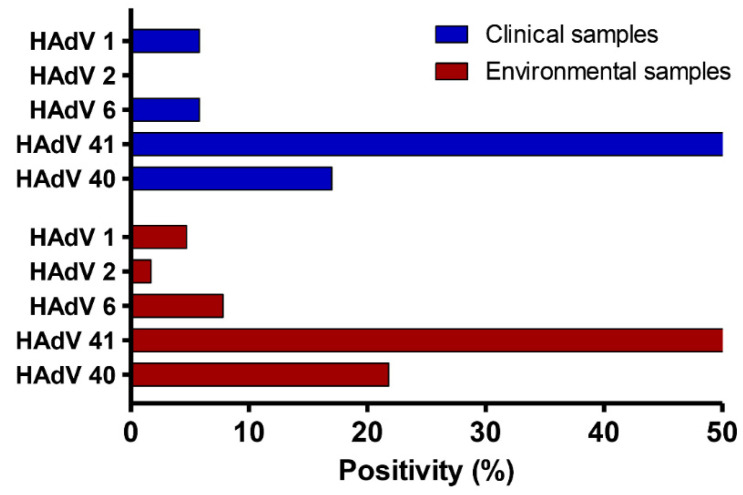
Distribution of HAdV genotypes in both fecal and sewage samples during a five-year period from 2016 to 2020.

During the 5-year investigation (Figure 3), three species of adenoviruses (B, C, and F) were identified, as well as five distinct genotypes. Species F exhibited the greatest incidence in fecal and sewage samples, at 88.5% and 85.9%, respectively. HAdV-41 was the most prevalent genotype [(71.2%) and (64%)], followed by HAdV-40 [(17.2%) and (21.8%)], HAdV-6 [(5.7%) and (7.8%)], HAdV-1 [(5.7%) and (4.7%)], and HAdV-2 [(0%) and (1.6%)] in fecal and sewage samples, respectively. Interestingly, the HAdV-41 genotype was the most common genotype throughout all the years of this study. 

The wastewater treatment facility, on the other hand, was successful in removing 57% (15/26) of adenovirus-41, 55% (5/9) of adenovirus 40, 25% (1/4) of adenovirus 6, and 100% of adenoviruses 1 and 2 (Table 4). 

Finally, when the hexon segments of the PCR-positive samples (*n* = 15) were sequenced and analyzed, they were found to be highly related to those of human adenoviruses in NCBI, namely Adv41, Adv40, Adv6, Adv1, and Adv2, with nucleotide similarity ranging between 97 and 100%. The most prevalent genotype throughout this study was human adenovirus 41.

### 3.5. Specific Cell Culture Isolation of Adenovirus-Positive Samples

Positive PCR samples, either fecal or sewage, were subjected to a cell culture isolation experiment. The symptoms of partial CPE appeared 96 h after infection. After 96 h, infected cells were rounding, detaching from plates, and partially clumping, but uninfected cells showed no such changes. Following that, a cell lysate was generated from the infected culture of the first passage in order to infect a freshly cultured cell line again. The second passage resulted in 100% cell death at 72 h post infection (data not shown). Finally, six out of ninety-nine samples exhibited a full CPE in HEp-2 cells throughout all passes. It should be noted that the isolated adenoviruses exhibited variable degrees of CPE, with some producing significant damage to the HEp-2 cell monolayer (most cells shrank and detached within 72–96 h post infection) and others generating just partial destruction. Some adenovirus types 40 and 41 (*n* = 6) were successfully propagated on HEp-2 cells, while the other species, identified by PCR, were not isolated by cell culture (Table 5).

## 4. Discussion

Human adenoviruses, after rotaviruses, are the second most common cause of diarrhea in infants in Egypt [10,11]. Adenovirus infections account for 2% to 10% of diarrheal cases [27]. Enteric HAdV can be transmitted through the fecal–oral route by contaminated water and food. Since they have been discovered in many types of water all year round and are more resistant to sewage treatment procedures, HAdV have been proposed as a suitable marker for emerging viral pollutants for human fecal contamination of water [8,12,13,28]. In general, people infected with enteric adenoviruses exhibit clinical symptoms such as diarrhea, vomiting, respiratory problems, and, in rare cases, acute disease or death. Adenovirus outbreaks are more likely to cause serious disease in infants and immune-compromised hosts, as well as in adults with respiratory or cardiac issues [3,9]. 

There is scant data on adenovirus epidemiology in Egypt, with most concentrating on enteric adenoviruses over short time periods (maximum two years) [12,13,14,15]. We investigated 5 years of detection, isolation, and genotyping of HAdV circulating in environment and clinical samples in Egypt from 2016 to 2020, to acquire a better knowledge of the incidence of HAdV infection through long-term investigation. The incidence rate of HAdV infections in fecal samples detected in this study was 7.8%, which is close to recent studies from Japan (4.8–7.9%) [29,30], China (9.8%) [31], India (11.8%) [32], Thailand (7.2%) [9], and Egypt (6.8%, and 2.7%), [12,13]. In comparison to other regions of the world, such as China, Poland, and Bangladesh [33,34,35], their detection rate of adenoviruses in fecal samples was up to 96.3%, which is much higher than our detection rate in the current study. These variations might be attributed to the diagnostic assays utilized or the geographical location of the collection area.

In the current study, the incidence of HAdV in environmental samples was 53.3%. In another study conducted in Egypt, sewage samples had a comparable high incidence (67%) [14]. In contrast, the incidence of HAdV was reported to be low in wastewater samples from Taiwan (27.3%) and Morocco (45.5%) [36,37]. In contrast, Brazil (100%) [38], South Africa (64%), Norway (92%), Greece (92.3%), and Poland (92.1%) were shown to have considerable HAdV prevalence [33,34,39,40]. The substantial discrepancy in these statistics among nations might be attributed to differences in wastewater treatment type, sampling site, and detection technology. Furthermore, our findings indicated that the wastewater treatment facility was ineffective in eradicating HAdV. As a consequence, the wastewater treatment facility was responsible for only a 36.7% drop in viral genome occurrence, which may be regarded as high risk because the wastewater treatment process was unable to considerably reduce the viral load. In any case, the presence of HAdV in ambient samples emphasizes the need for waterborne virus surveillance.

We, on the other hand, investigated the incidence of adenoviral infection in children of all ages and genders. According to our 5-year study, male and female positive detection rates were comparable and did not differ substantially (*p =* 0.19). Children under the age of two made up 77.1% (27/35) of those afflicted, a statistically significant difference (*p* < 0.001) that was consistent with earlier studies [2,10,12,14]. 

In terms of seasonal distribution, the major peak time for adenovirus infection is still a point of contention. Our findings showed that HAdV have a peaked time for clinical samples from December to February (*p* < 0.001), which corresponded to Egypt’s rainy season. There was no statistically significant season trend in the monthly distribution of HAdV in sewage samples. Our findings are consistent with earlier research from Egypt [12,13,14], Thailand [9], and India [32]. This data adds to the epidemiologic picture of HAdV and might be valuable for preventing and managing HAdV in children, as well as future vaccine development.

HAdV was detected in this study’s fecal samples at lower rates in 2020 (3.9%) and 2016 (5.8%), with the highest frequency rate in 2018 (12.8%). While HAdV was found in sewage samples at a lower rate in 2016 (37.5%), the highest frequency rates were in 2019 (66.5%), 2018 (62.5%), and 2020 (54.1%). The co-circulation of enteric and non-enteric viruses might explain the high frequency rate in fecal or sewage samples. Notably, we observed that the highest frequencies of adenoviruses were detected mostly in sewage samples, owing to the diversity of populations observed in sewage. Furthermore, HAdV-type 41 had the highest genotype frequency in both feces (71.2%) and sewage (64%) samples in the present study. This conclusion is consistent with previous epidemiological studies conducted in Egypt, Thailand, and China, which discovered that HAdV-41 was the most often recognized genotype in children with gastroenteritis and sewage samples, with rates ranging from 22% to 60% [2,9,12,13,14,15]. According to prior studies, gastroenteritis has also been linked to non-enteric adenoviruses [9]. Individuals with acute gastroenteritis were found to have HAdV-C1, C2, C5, and B3 genotypes [9,35,41]. Non-enteric adenovirus genotypes Ad-C1 (5%), Ad-C2 (1.6%), and Ad-6 (7%) were reported in this study. As a result, our data support that non-enteric adenoviruses might play a role in diarrheal disease. 

In terms of adenovirus isolation, the HEp-2 cell line successfully supports viral isolation and proliferation. Our findings were consistent with previous research conducted in 1988 and 2002 [25,42], which proved the use of HEp-2 cells for the isolation and propagation of human enteric adenoviruses. Six of the ninety-nine positive fecal and sewage adenovirus samples were successfully recovered, and developed a CPE in all repeated passing, indicating complete viral replication. All six isolated samples were HAdV 41 and 40, which was consistent with previous findings that supported utilizing the HEp-2 cell line to isolate enteric and non-enteric adenovirus types [25,42]. Our future plans include whole-genome sequencing of successfully isolated adenovirus types to track all alterations in such genomes, as well as attempting to use these isolated viral candidates to develop a preventive vaccine against adenovirus infections in order to overcome the issue in Egypt. This study also highlights the necessity to continuously monitor circulating HAdV, and to improve current wastewater treatment methods to ensure the quality of the effluents discharged upon wastewater treatment into the environment, threatening public health [1,43]. 

## 5. Conclusions

This study used qPCR to examine 447 fecal specimens of children hospitalized with diarrhea and 120 samples from sewage, collected over a 5-year period from 2016–2020, for the presence of human adenoviruses. HAdV-F40 and HAdV-F41 account for the greatest proportion of the adenoviruses identified, showing the importance of these types as causative agents of diarrhea in young children in Cairo, Egypt. 

## Figures and Tables

**Table 1:** Incidence of HAdV in children with acute diarrhea in Cairo, Egypt from 2016 to 2020.

Year	No. of Samples	Positive Samples Number, (%)	% of Positive Samples	
			Female	Male
2016	69	4 (5.8)	2/30 (6.6)	2/39 (5.1)
2017	90	7 (7.7)	1/38 (2.6)	6/52 (11.5)
2018	101	13 (12.8)	5/47 (10.6)	8/54 (14.8)
2019	110	8 (7.3)	3/41 (7.3)	5/69 (7.2)
2020	77	3 (3.9)	0/33 (0)	3/44 (6.8)
5 years	447	35 (7.83)	11/189 (5.8)	24/258 (9.3)

**Table 2:** Distribution of the adenovirus’ positivity in children according to age groups.

Age (Months)	2016		2017		2018		2019		2020		Total
	M *	F **	M	F	M	F	M	F	M	F	
<12	2/12 (16.6)	1/10 (10)	3/21 (25)	1/17 (5.8)	2/16 (12.5)	2/15 (13.3)	1/30 (3.3)	1/12 (8.3)	2/21 (9.5)	0/3 (0)	15/157 (9.5)
12 ≤ 24	0/11 (0)	1/8 (12.5)	1/12 (8.3)	0/9 (0)	3/11 (27.2)	3/13 (23)	1/14 (7.1)	2/9 (22)	1/20 (5)	0/21 (0)	12/128 (9.3)
24 ≤ 36	0/10 (0)	0/5 (0)	1/8 (12.5)	0/12 (0)	1/14 (7.1)	0/7 (0)	2/9 (22)	0/3 (0)	NA	0/5 (0)	4/73 (5.4)
36 ≤ 48	0/3 (0)	0/7 (0)	1/11 (9)	NA	1/7 (14.2)	0/5 (0)	1/7 (14)	0/5	0/3 (0)	0/2 (0)	3/50 (6)
48 ≤ 60	0/3 (0)	NA	NA	NA	1/6 (16.6)	0/7 (0)	0/9 (0)	0/12 (0)	NA	0/2 (0)	1/39 (2.5)
Total	2/39 (5.2)	2/30 (6.6)	6/52 (11.5)	1/38 (2.6)	8/54 (14.8)	5/47 (10.6)	5/69 (7.2)	3/41 (7.3)	3/44 (6.8)	0/33 (0)	35/447 (7.8)

M * refers to male, while F ** refers to female, but NA is meaning not applicable.

**Table 3:** Incidence of HAdV in sewage samples in Egypt from 2016 to 2020.

Year	No. of Samples	Positive Samples Number, (%)	% of Positive Samples	
			Inlet	Outlet
2016	24	9 (37.5)	7/12 (58.3)	2/12 (16.6)
2017	24	11 (45.8)	6/12 (50)	5/12 (41.6)
2018	24	15 (62.5)	11/12 (91.6)	4/12 (33.3)
2019	24	16 (66.6)	9/12 (75)	7/12 (58.3)
2020	24	13 (54.1)	10/12 (83.3)	3/12 (25)
5 years	120	64 (53.3)	43/60 (71.7)	21/60 (35)

**Table 4:** Environmental positive HAdV genotypes distribution in raw and treated samples from 2016 to 2020.

Genotypes	2016		2017		2018		2019		2020		Total
	Raw	Treated	Raw	Treated	Raw	Treated	Raw	Treated	Raw	Treated	
Adenovirus-41	4	2	3	3	5	2	6	5	8	3	41
Adenovirus-40	2	0	1	1	3	2	2	2	1	0	14
Adenovirus-6	0	0	1	1	1	0	1	0	1	0	5
Adenovirus-2	0	0	0	0	1	0	0	0	0	0	1
Adenovirus-1	1	0	1	0	1	0	0	0	0	0	3
Total	7	2	6	5	11	4	9	7	10	3	64

**Table 5:** The description of isolated samples by cell culture assay.

Strain Name	Source of Isolation	Year of Collection	Titration by qPCR	Isolated Genotype	Isolation Passages		
					1st Passage	2nd Passage	3rd Passage
AMDT-148	Sewage, Inlet	2017	2.5 × 10^4^	Ad-41	*	**	**
AMDT-149	Stool	2018	1.4 × 10^6^	Ad-41	**	**	**
AMDT-021	Stool	2019	2.7 × 10^5^	Ad-40	*	**	**
AMDT-014	Sewage, Inlet	2018	3.8 × 10^3^	Ad-41	*	*	**
AMDT-057	Stool	2019	1.6 × 10^4^	Ad-40	*	*	**
AMDT-201	Stool	2019	1.1 × 10^6^	Ad-41	**	**	**

* means partial CPE, and ** means Full CPE.

## Data Availability

Not applicable.

## Supplementary Materials

The following supporting information can be downloaded at: https://www.mdpi.com/article/10.3390/v14102192/s1, Figure S1: Development of Cytopathic Effect on HEp-2 cell line during isolation of adenoviruses.
