# Supplementary material for: Isolation and Genotyping of Adenoviruses from Wastewater and Diarrheal Samples in Egypt from 2016 to 2020
Source: Viruses. 2022 Oct 4;14(10):2192. doi: 10.3390/v14102192 (PMC9609437; doi:10.3390/v14102192)
Supplement: Supplementary file 1 [file viruses-14-02192-s001.zip › viruses-1919659-supplementary.pdf]

Isolation and Genotyping of Adenoviruses from Wastewater and Diarrheal Samples in Egypt from 2016 to 2020

Abdou Kamal Allayeh[1,*], Sahar Abd Al-Daim[1], Nehal Ahmed[1], Mona El-Gayar[2] and Ahmed Mostafa[3,*]

[1] Virology Lab 176, Water Pollution Research Department, Environment and Climate Change Institute, National Research Centre, Dokki, Giza 12622, Egypt
[2] Microbiology Department, Faculty of Pharmacy, Ain Shams University, El-Qobba Bridge, El Weili 11566, Egypt
[3] Center of Scientific Excellence for Influenza Viruses, National Research Centre, Dokki, Giza 12622, Egypt
* Correspondence: drallayeh@yahoo.com (A.A.); ahmed_elsayed@daad-alumni.de (A.M.)

Supplementary Figure S1

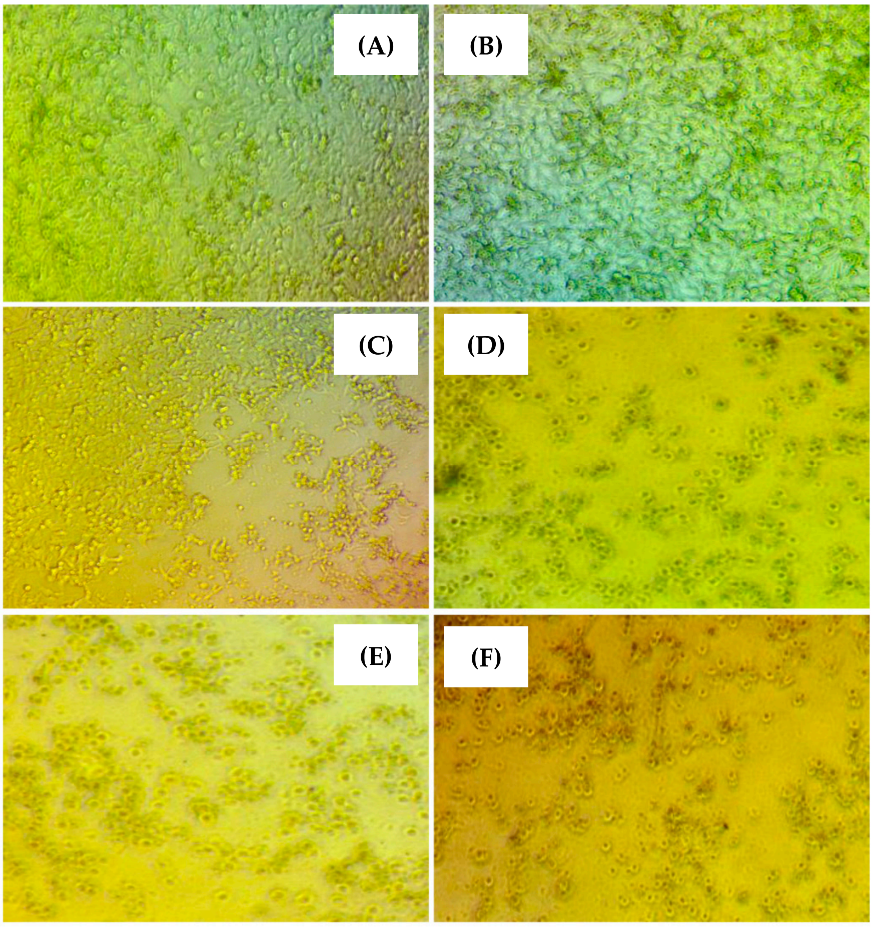

Figure S1. Development of Cytopathic Effect on Hep-2 Cell line during isolation of Adenoviruses; (**A**,**B**) Control HEp-2 cell line after 4 days of propagation; (**C**) Infected Hep-2 by positive sample of Adenovirus showing partial CPE; (**D**–**F**) Infected Hep-2 cells by positive samples of Adenovirus showing complete CPE.
